# Eco-friendly synthesis of metal dichalcogenides nanosheets and their environmental remediation potential driven by visible light

**DOI:** 10.1038/srep15718

**Published:** 2015-10-27

**Authors:** Ashish Kumar Mishra, K. V. Lakshmi, Liping Huang

**Affiliations:** 1 Department of Materials Science and Engineering, Rensselaer Polytechnic Institute, Troy, NY, USA-12180; 2 Department of Chemistry and Chemical Biology, Rensselaer Polytechnic Institute, Troy, NY, USA-12180

## Abstract

Exfoliated transition metal dichalcogenides (TMDs) such as WS_2_ and MoS_2_ have shown exciting potential for energy storage, catalysis and optoelectronics. So far, solution based methods for scalable production of few-layer TMDs usually involve the use of organic solvents or dangerous chemicals. Here, we report an eco-friendly method for facile synthesis of few-layer WS_2_ and MoS_2_ nanosheets using dilute aqueous solution of household detergent. Short time sonication of varying amount of bulk samples in soapy water was used to scale up the production of nanosheets. Thermal stability, optical absorption and Raman spectra of as-synthesized WS_2_ and MoS_2_ nanosheets are in close agreement with those from other synthesis techniques. Efficient photocatalytic activity of TMDs nanosheets was demonstrated by decomposing Brilliant Green dye in aqueous solution under visible light irradiation. Our study shows the great potential of TMDs nanosheets for environmental remediation by degrading toxic industrial chemicals in wastewater using sunlight.

Two-dimensional (2D) transition metal dichalcogenides (TMDs), such as WS_2_ and MoS_2_, have been extensively studied due to their unique catalytic, optical and electronic properties^1^,^2^,^3^,^4^,^5^,^6^,^7^,^8^. Quantum confinement of charge carriers in nanostructured TMDs have been exploited in energy storage, catalysis, sensing and optoelectronic devices^9^,^10^,^11^. Noticeably, the band gap of nano-MoS_2_ increases substantially from the bulk value of ~1.2 eV to ~1.8 eV, giving rise to large blueshifts in its absorption edges relative to that of the bulk counterpart. Shifts in the conduction and valence band edges of nano-MoS_2_ lead to appropriate changes in the redox potentials, which allow it to act as a visible light responsive photocatalyst^12^,^13^,^14^,^15^,^16^,^17^,^18^,^19^. Moreover, the electronic states of the conduction and valence bands both primarily come from Mo 4d orbitals, so photo-excitation of electrons would not significantly weaken bonds between Mo and S atoms, leading to enhanced photochemical stability^13^. Therefore, it is possible to exploit sunlight as the sole energy source required for cleanup of harmful chemicals from the environment by tapping into the great photocatalytic potential of nanostructured TMDs, if available in large quantity with low cost. Visible light responsive photocatalysts based on nanostructured TMDs will find a broader range of applications, comparing with the traditional photocatalysts, such as TiO_2_ and ZnO with a wide band gap (>3 eV), which can only act under UV light irradiation.

Different techniques like mechanical exfoliation, chemical vapour deposition and liquid exfoliation have been investigated for synthesizing TMDs nanosheets. Mechanical exfoliation^9^,^10^,^11^ and chemical vapour deposition methods result in high quality but low yield of nanosheets generally suited for electronic applications^20^,^21^. Liquid processed samples result in greater yield of few-layer nanomaterials and can be applied for energy storage and catalytic purposes. Ion intercalation and sonication assisted exfoliation have been used in liquid process technique^22^,^23^,^24^. Among intercalation approaches, one method involves the intercalation of lithium ions in the interlayer space of the bulk material, followed by immersing the resulting compound in water. The lithium ions react with water to form LiOH and H_2_, which expands the interlayer space, causing the bulk material to exfoliate into 2D nanomaterials^3^,^24^,^25^. This process involves the use of highly flammable and explosive chemicals. Alternatively, electrochemical intercalation of lithium ions avoids the use of dangerous chemicals but unfortunately suffers with low yields, thus defeating the purpose^26^,^27^. In another liquid phase exfoliation method, few-layer materials are exfoliated from the bulk material using ultrasound waves (sonication), while dispersed in polar solvents, with and without the aid of surfactants^24^,^25^. Physical properties of solvent such as boiling point, surface tension and energy, as well as solubility parameters affect the resulting 2D nanomaterial^28^,^29^,^30^. Majority of sonication methods involve use of organic solvents like N-methylpyrrolidone (NMP), mixture of ethanol and water, benzene, acetone, or organic compounds like iodixanol and Pluronic F68^7^,^25^,^29^. Large-scale, cost-effective and environmentally friendly synthesis of TMDs nanosheets remains a great challenge.

Here, we report a facile technique by using the sonication process in a mixture of household detergent (Ultra Tide) and water for the large-scale synthesis of few-layer WS_2_ and MoS_2_ nanosheets. Our study demonstrated that sophisticated surfactants are not necessary to exfoliate TMDs, dilute aqueous solution of simple household detergent works extremely well at a very low cost (~0.01 cent per batch of synthesis in this study), without the need of dangerous chemicals or harsh synthesis conditions, which are key to eco-friendly large-scale manufacturing of few-layer TMDs nanosheets.

Photocatalytic effect of few-layer WS_2_ and MoS_2_ nanosheets was demonstrated by decomposing Brilliant Green (BG) dye in aqueous solution under visible light irradiation. BG (ammonium, 4-(p-diethylamino)-alpha-(phenylbenzylidene), C_27_H_34_N_2_O_4_S) being extensively used in textile dying and paper printing, is a common organic chemical in industrial wastewater. It is carcinogenic in nature and considered highly toxic for humans and animals due to possible cause of eyes injury, irritation to the respiratory and gastrointestinal tract^31^,^32^,^33^,^34^. Our study shows that WS_2_ and MoS_2_ nanosheets have great potential in environmental remediation by utilizing sunlight to degrade harmful chemicals such as BG in wastewater.

## Results

### Production of Nanosheets

**Figure 1:**
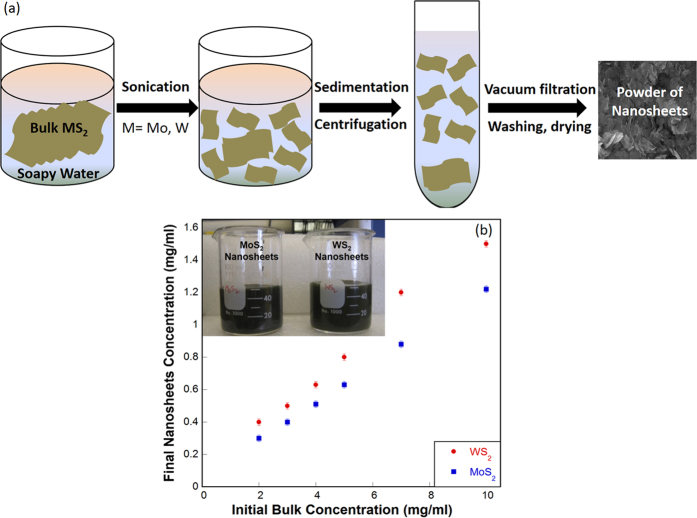
Production of WS_2_ and MoS_2_ nanosheets. (**a**) Schematic illustration of the synthesis process for WS_2_ and MoS_2_ nanosheets (drawn by the authors). (**b**) Resultant concentration of as-synthesized WS_2_ and MoS_2_ nanosheets as a function of the initial bulk concentration in 50 ml of di water mixed with 25 mg of detergent (Ultra Tide). Inset image shows the dispersed solutions containing WS_2_ and MoS_2_ nanosheets.

Figure 1 shows the resultant concentration of as-synthesized WS_2_ and MoS_2_ nanosheets as a function of their initial bulk concentration used in sonication. In this method, varying amount of bulk WS_2_ and MoS_2_ (2, 3, 4, 5, 7 & 10 mg/ml) were first stirred in 50 ml di water (at 40–50 °C) and 25 mg of detergent (Ultra Tide) for 20 min at 300 rpm, followed by 3 h sonication using a solid probe sonicator. Similar to the effect of surfactant^35^, Ultra Tide detergent reduces the surface tension of water and induces better solubility of bulk WS_2_ and MoS_2_^23^,^29^. Schematic illustration of the synthesis process is shown in Fig. 1a. High intensive ultrasound waves help peel few-layer nanosheets off the bulk sample. Nanosheets dispersed solution was obtained by sedimentation and centrifugation at 1000 rpm for 30 min after sonication. Dispersed solutions (inset image – Fig. 1b) were found stable for several weeks.

Increasing amount of initial bulk sample results in higher concentration of well dispersed nanosheets. Figure 1b indicates a higher production rate of WS_2_ nanosheets compared to MoS_2_ nanosheets under similar conditions. This can be directly attributed to the better dispersion of bulk WS_2_ in soapy water compared to bulk MoS_2_. Maximum concentration of 1.50 ± 0.02 mg/ml for WS_2_ nanosheets and 1.22 ± 0.02 mg/ml for MoS_2_ nanosheets were obtained with initial bulk concentration of 10 mg/ml in 50 ml di water mixed with detergent. These concentrations were measured by different batches of solutions with repeated experiments of vacuum filtration and washing followed by drying and weighing the filter. In a single experiment, nearly 76 and 62 mg of WS_2_ and MoS_2_ nanosheets were prepared, respectively. These yields are comparable to that could be obtained in the large-scale liquid exfoliation synthesis method that the Coleman group used, which gave 3.2 mg/ml of MoS_2_ nanosheets in 20 ml NMP after 23 h of sonication, i.e., 64 mg of nanosheets per batch^36^.

### Electron Microscopy Analysis.

**Figure 2:**
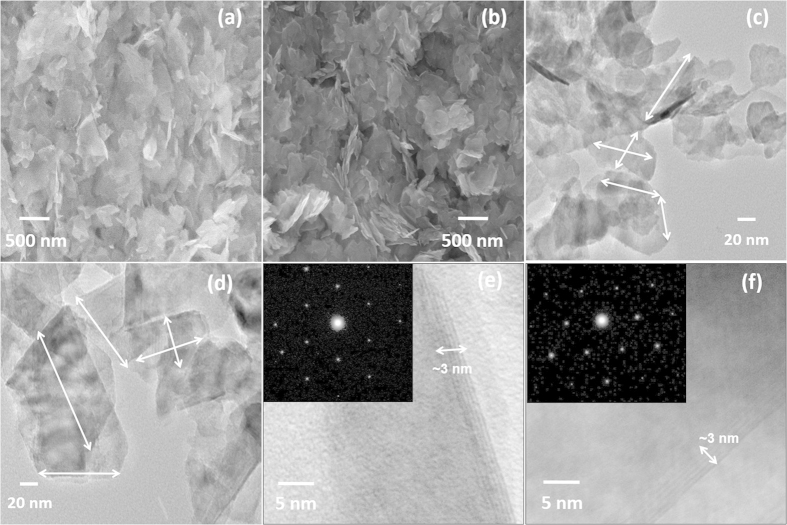
Structure of WS_2_ and MoS_2_ nanosheets. SEM images of (**a**) WS_2_ and (**b**) MoS_2_ nanosheets. TEM images of WS_2_ (**c,e**) and MoS_2_ (**d,f**) nanosheets. Inset images in (**e**,**f**) show the corresponding electron diffraction pattern of WS_2_ and MoS_2_ nanosheets, respectively.

Structural analysis of as-synthesized WS_2_ and MoS_2_ nanosheets was performed using electron microscopy techniques. Morphologies in scanning electron microscopy (SEM) images in Fig. 2a,b show that WS_2_ and MoS_2_ nanosheets are uniformly distributed and differently oriented. Figure 2c,d show transmission electron microscopy (TEM) images of WS_2_ and MoS_2_ nanosheets at low magnification, respectively, indicating thin layered structures with lateral dimension distributions in the range of 60–90 nm for WS_2_ and 80–120 nm for MoS_2_, respectively. To observe the height distribution of as-synthesized nanosheets, edge views of nanosheets were focused at a small tilt angle (~8–10°). Figure 2e,f clearly indicate the height distribution of nanosheets around 3 nm, corresponding to 4–6 layers of nanosheets for WS_2_ and MoS_2_, respectively. Selective area electron diffraction patterns in the inset images in Fig. 2e,f indicate the in-plane hexagonal atomic arrangements in as-synthesized WS_2_ and MoS_2_ nanosheets.

### Optical Characterization

**Figure 3:**
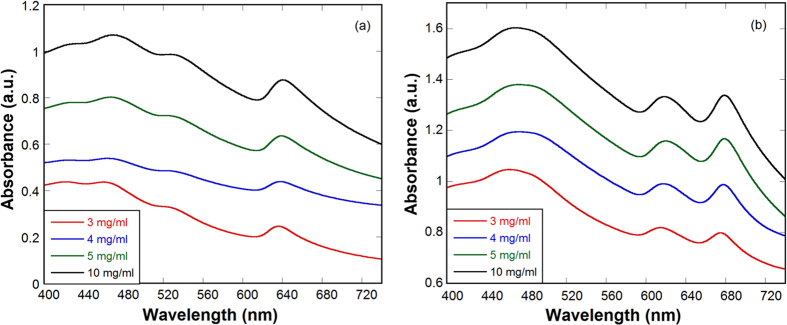
Characterization by UV-Visible spectroscopy. Absorbance spectra of (**a**) WS_2_ and (**b**) MoS_2_ nanosheets, prepared with different initial concentration of bulk samples indicated in legends.

As-synthesized WS_2_ and MoS_2_ nanosheets were characterized by UV-visible absorption and Raman spectroscopies. To remove the effect of detergent in the absorbance spectra, nanosheets dispersed solutions were filtered and washed twice. Powder samples of WS_2_ and MoS_2_ nanosheets were re-dispersed in di water to take absorption spectra. Figure 3a,b show that absorption spectra of WS_2_ and MoS_2_ nanosheets prepared with different initial bulk concentrations exhibit similar peak positions for each material, indicating nearly the same thickness distribution of nanosheets irrespective of the initial bulk concentration.

Excitonic absorption peaks arising from the direct gap transitions at the K point of the Brillouin zone correspond to the bands at 530 and 639 nm for WS_2_, 617 and 679 nm for MoS_2_ nanosheets. Additional peaks around 460 nm are attributed to the optical transitions between the density of states peaks in the valence and conduction bands^37^,^38^,^39^. Figure 3 is in good agreement with reported absorption spectra of nanosheets synthesized via other sonication methods^25^,^29^. The first peak at long wavelength in Fig. 3a,b correspond to the lowest optical band gap of ~1.9 and ~1.8 eV for WS_2_ and MoS_2_ nanosheets, higher than the corresponding bulk value of ~1.4 and ~1.2 eV, respectively. Increase in band gap is a clear indication of the quantum confinement in nanosheets and the above values for few-layer WS_2_ and MoS_2_ are close to those of single-layer counterparts^1^.

**Figure 4:**
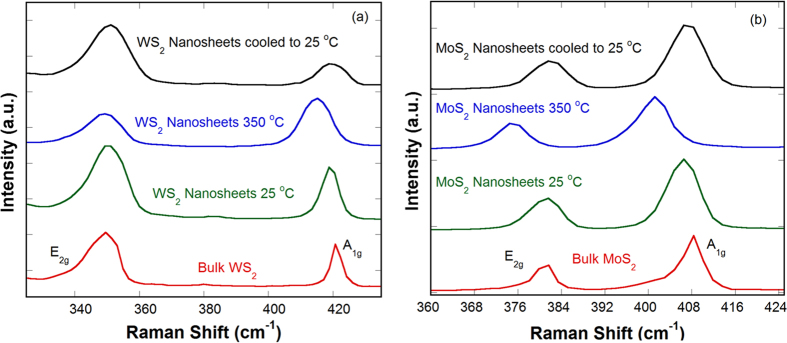
Characterization by Raman spectroscopy. Raman spectra for (**a**) WS_2_ and **(b**) MoS_2_.

Raman spectra of washed and dried WS_2_ and MoS_2_ nanosheets were taken to show their in-plane 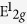 and out-of-plane A_1g_ modes. Temperature dependent Raman spectroscopy was used to study their thermal stability and temperature coefficients of peak positions were also calculated. Figure 4a shows the 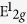 and A_1g_ modes are at around 349.6 and 420.6 cm^−1^ for bulk WS_2_ at room temperature (25 °C). The corresponding modes in WS_2_ nanosheets occur at around 350.2 and 419.1 cm^−1^ at room temperature. Upon heating to 350 °C, WS_2_ nanosheets show red shifts in Raman peaks (~349.0 and 415.0 cm^−1^). Raman spectrum of cooled WS_2_ nanosheets shows that the peak positions (~350.5 and 419.2 cm^−1^) approach to their pristine values. Similar behaviors are observed for MoS_2_ nanosheets in Fig. 4b. The red shifts in Raman peaks are more obvious in MoS_2_ nanosheets upon heating from room temperature (~381.4 and 406.6 cm^−1^) to 350 °C (~375.3 and 401.2 cm^−1^). Energy difference of 25.2 cm^−1^ between 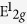 and A_1g_ modes at room temperature corresponds to 5–6 layer of MoS_2_ nanosheets as described in other reports^40^, which is in good agreement with our TEM analysis.

Vibration of the 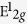 mode involves the in-plane opposing motions of S and W/Mo atoms, and that of A_1g_ mode is the out-of-plane relative motions of S atoms^40^,^41^. As the temperature increases, both of the Raman-active modes soften linearly and the A_1g_ peak clearly broadens. The evolution of the Raman peak position ω (in cm^−1^ units) as a function of lattice temperature follows a linear dependence (Fig. S1). The temperature coefficient of −0.012 cm^−1^ K^−1^ and −0.014 cm^−1^ K^−1^ were obtained for the A_1g_ peak of WS_2_ and MoS_2_ nanosheets, respectively, which are in good agreement with other reports^42^,^43^,^44^. Detail calculations are given in Supplementary section S1.

### Visible Light Responsive Photocatalytic Activity

**Figure 5:**
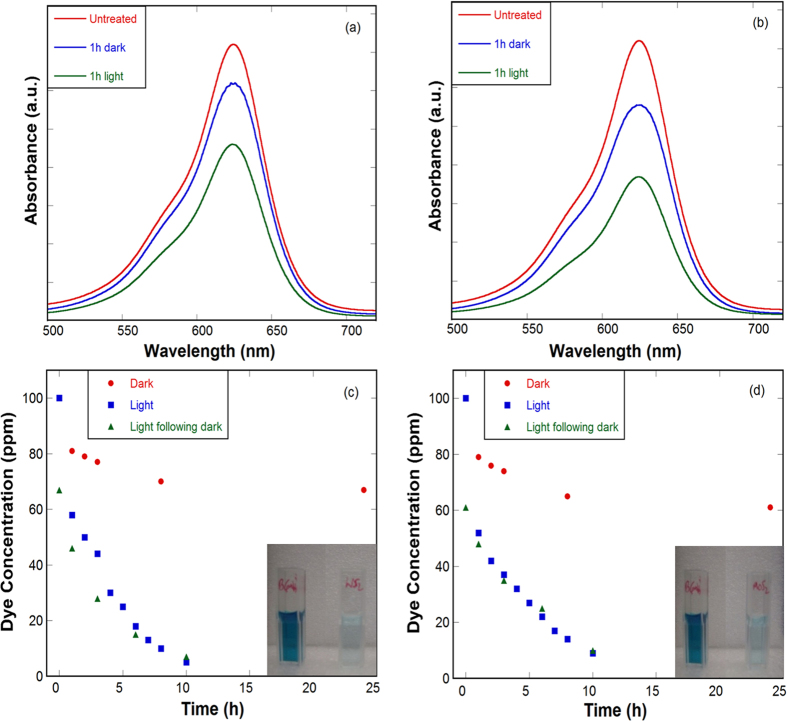
Photocatalytic effect of WS_2_ and MoS_2_ nanosheets. Absorbance spectra and concentration variation of BG solution treated with (**a,c**) WS_2_ and (**b,d**) MoS_2_ nanosheets. Inset images show the corresponding untreated and treated samples.

Photocatalytic activity of WS_2_ and MoS_2_ nanosheets was tested by decomposition of Brilliant Green (BG) dye under visible light irradiation. In this process, 5 ml of 100 ppm of BG aqueous solution was treated with 1.4 mg of nanosheets in dark and under white light illumination without stirring or sonication. Intensity of the absorption peak of BG at ~625 nm was used to track its concentration in the solution. Figure 5a,b show the absorption spectra of untreated and treated BG solution with WS_2_ and MoS_2_ nanosheets, respectively, indicating that visible light assisted BG reduction is much faster than treatment in dark. There is no noticeable concentration change in BG solution under white light irradiation for the same amount of time without WS_2_ and MoS_2_ nanosheets. This clearly demonstrates the photocatalytic effect of WS_2_ and MoS_2_ nanosheets.

Figure 5c,d show the time dependent BG reduction kinetics in treatment with WS_2_ and MoS_2_ nanosheets, respectively. Surface area for WS_2_ and MoS_2_ nanosheets were measured by BET to be around 59 and 58 m^2^ g^−1^, respectively. In dark physical adsorption is responsible for BG reduction, while under visible light irradiation photocatalytic decomposition takes place. It was observed that the physical adsorption mainly takes place within 1 h and reaches saturation in 24 h treatment in dark, resulting in nearly 33 and 39% of BG reduction with WS_2_ and MoS_2_ nanosheets, respectively. After 10 h visible light irradiation, nearly 95 and 91% BG reduction was observed with WS_2_ and MoS_2_ nanosheets, respectively, with and without previous treatment in dark. Major reduction of nearly 60 and 75% occur within 3 and 5 h, respectively, for both nanosheets. During the decomposition process, sedimentation of nanosheets at the bottom takes place. Active stirring/sonication of solution under visible light irradiation may facilitate the contact between BG and nanosheets, further increasing the decomposition rates.

As-synthesized WS_2_ and MoS_2_ nanosheets show nearly 60% reduction of 100 ppm BG solution in 3 h under illumination of two 22 W circline visible light fluorescence lamps fitted in a ring illuminator. Aqueous solution of Degussa P-25 TiO_2_ nanoparticles (~21 nm in size, 80% anatase and 20% rutile) shows nearly 60% reduction of 50 ppm BG solution under UV illumination (two 15 W UV-365 nm lamps) for 4 h with comparable catalyst concentration^31^. Comparable concentration of Nb_2_O_5_ gives nearly 55% reduction of 10 ppm BG solution in 200 min (~3.3 h) under 500 W UV illumination^32^. Photodegradation of BG under visible light irradiation follows first-order kinetics as shown in the Supplementary section S2. The photocatalytic reaction rate constants were found to be 3.67 × 10^−3^ and 2.83 × 10^−3^ min^−1^ for WS_2_ and MoS_2_ nanosheets, respectively. These rates are comparable to that of 500 W UV light assisted decomposition of BG with Nb_2_O_5_ (i.e., ~3 × 10^−3^ min^−1^) using comparable concentration of catalyst^32^. The above observations clearly show that WS_2_ and MoS_2_ nanosheets are efficient photocatalysts for decomposing organic chemicals such as BG without the need of UV irradiation.

The photocatalytic process involves the generation of electron-hole pairs in TMDs nanosheets (band gap <2 eV) under visible light irradiation. Due to the quantum confinement effect in TMDs nanosheets, the conduction and valence band edges change such that the oxidation potential is large enough to produce hydroxyl radicals (**˙**OH), and the reduction potential is suitable for the formation of superoxide radical anions (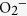), both acting as the oxidizing species in the photocatalytic processes^31^. Most of the **˙**OH radicals are generated from the reaction between the holes and surface-adsorbed H_2_O or 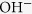. The probability for the formation of 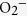 is less than that of **˙**OH species^31^. The *N*-de-ethylation of the BG dye occurs mostly by the attack of the **˙**OH species on the *N*,*N*-diethyl groups of the BG dye, resulting in stepwise photochemical process to generate mono-, di-, tri-, and tetra-*N*-de-ethylated BG species at intermediate steps and lead to CO_2_ and water as final products^31^,^32^,^33^,^34^. It is also reasonable to believe that photogenerated electron-hole pairs have a much shorter distance to travel to surfaces/edges in TMDs nanosheets, participating in redox reactions before recombination, hence enhancing the photocatalytic activity.

## Discussion

In conclusion, a facile and eco-friendly technique has been developed for the scalable synthesis of few-layer WS_2_ and MoS_2_ nanosheets. Similar to the effect of surfactant^35^, household detergent reduces the surface tension of water, and ultrasonic waves help peel off the few-layer nanosheets from the bulk materials. Formation of nanosheets in the solution was confirmed using electron microscopy and optical spectroscopy techniques. Their thermal stability and temperature coefficients of peak shifts were examined using *in-situ* Raman spectroscopy. Visible light responsive photocatalytic efficiency of WS_2_ and MoS_2_ nanosheets for BG decomposition demonstrates their great potential for degrading toxic industrial chemicals in wastewater using sunlight.

## Methods

### Synthesis of Nanosheets

Bulk WS_2_ and MoS_2_ (both from Sigma Aldrich) were stirred separately in 50 ml di water mixed with 25 mg of house-hold detergent (Ultra Tide) for 20–30 min at 40–50 °C. Thereafter the solutions were sonicated for 3 h by solid probe sonicator (Sonics Vibra Cell Sonicator VC 750- 1/8″ tapered micro tip, 750 W maximum power and 20 kHz frequency) using 30% amplitude of power with 6 s on and off pulses. The resultant solutions contain TMDs nanosheets, micron thick sheets and residue bulk materials. The solutions were allowed to sediment for 3 h to remove the residue bulk and further centrifuged at 1000 rpm for 30 min to remove the thick sheets. Resultant solutions contained well dispersed WS_2_ and MoS_2_ nanosheets. Powder samples were obtained by vacuum filtration and repeated washing, followed by drying in air. Resultant concentrations of the nanosheets dispersed solutions were measured by weighing the filter before and after filtration and drying.

### Characterization

Characterization of as-synthesized nanosheets was performed using microscopy and spectroscopy techniques. Washed and dried powder samples were used for characterization. Structural analysis was performed using a JEM 2010 transmission electron microscope (TEM) at 200 kV. TEM analysis was performed by depositing WS_2_ and MoS_2_ nanosheets dispersed solutions in isopropyl alcohol over the carbon coated copper grid followed by drying in air. Surface morphology of WS_2_ and MoS_2_ nanosheets was studied using a JSM 6335 field emission scanning electron microscope (SEM) at 20 kV. Powder samples were deposited over carbon tape for SEM study. Absorption spectra of WS_2_ and MoS_2_ nanosheets re-dispersed in di water were taken by a Perkin Elmer Lambda950 UV-visible spectrophotometer with step size of 1 nm. Spectroscopy analysis of powder samples was also performed using a LabRAM HR800 Raman microscope with 20 mW power of a 532 nm green laser and a 100x objective to give a spatial resolution of ~1 μm.

### Photocatalysis

Concentration of BG dye in solution was measured using a Perkin Elmer UV-visible absorption spectrophotometer. Visible light illumination was created using a Ring illuminator (Luzchem Research, Inc.), fitted with two 22 W, 8″ Circline Fluorescent lamps. BG solutions with WS_2_ and MoS_2_ nanosheets were kept in bottle in the center of the illuminator ring. After treatment with nanosheets, solutions were centrifuged for one minute at 5000 rpm to separate the nanosheets and then used to measure the absorption spectra for calculating the residue dye concentration in solution.

## Additional Information

**How to cite this article**: Mishra, A. K. *et al.* Eco-friendly synthesis of metal dichalcogenides nanosheets and their environmental remediation potential driven by visible light. *Sci. Rep.* **5**, 15718; doi: 10.1038/srep15718 (2015).

## Supplementary Material

Supplementary Information
