# Supplementary material for: Eco-friendly synthesis of metal dichalcogenides nanosheets and their environmental remediation potential driven by visible light
Source: Sci Rep. 2015 Oct 27;5:15718. doi: 10.1038/srep15718 (PMC4621539; doi:10.1038/srep15718)
Supplement: Supplementary Information [file srep15718-s1.pdf]

SUPPLEMENTARY INFORMATION

Eco-friendly synthesis of metal dichalcogenides nanosheets and their environmental remediation potential driven by visible light

Ashish Kumar Mishra[a], K.V. Lakshmi[b] and Liping Huang[a*]

[a]Department of Materials Science and Engineering, Rensselaer Polytechnic Institute, Troy, NY, USA-12180

[b]Department of Chemistry and Chemical Biology, Rensselaer Polytechnic Institute, Troy, NY, USA-12180

S1. Temperature dependent Raman Spectra

Raman spectra of WS_2 and MoS_2 nanosheets were taken from room temperature to 623 K with a heating rate of 20 K/min in air using a Linkam TH600 heating stage. Shifts in the A_{1g} and E^1_{2g} modes as a function of temperature were observed in WS_2 and MoS_2 nanosheets as seen in Figure S1, which indicates a linear variation for both nanomaterials.

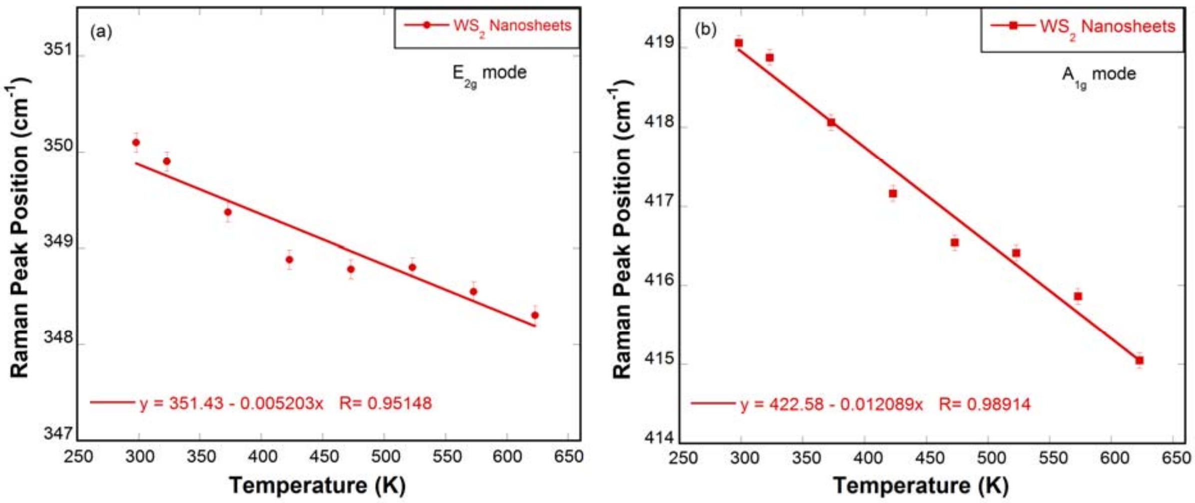

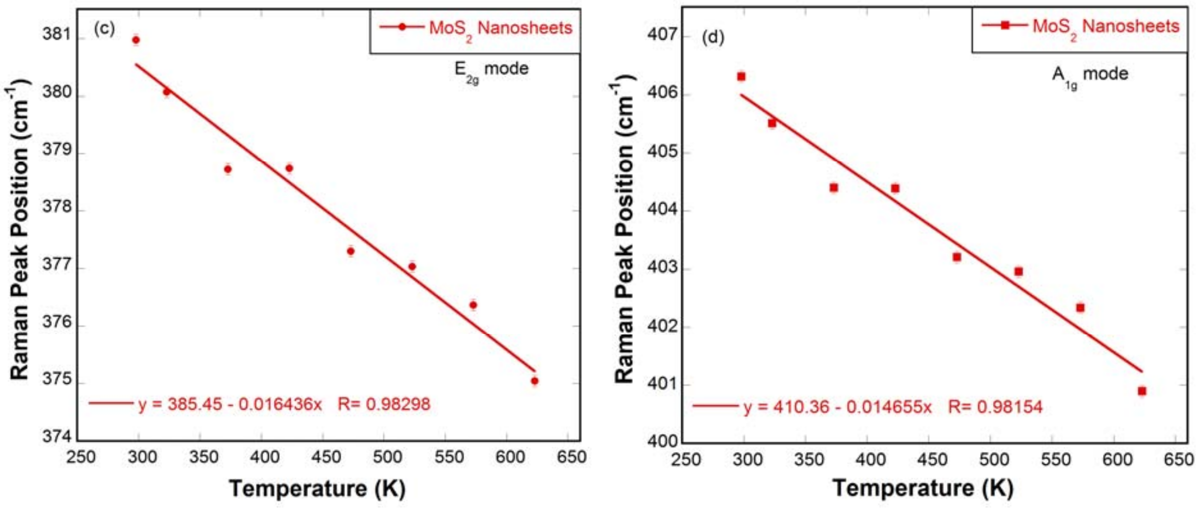

Figure S1 | Temperature dependent Raman spectroscopy study. Temperature dependent Raman shift in E_{2g} and A_{1g} modes of (a, b) WS_2 and (c, d) MoS_2 nanosheets.

Temperature dependent peak positions of E^1_{2g} and A_{1g} modes were fitted using following equation:

$$\omega(T) = \omega_0 + \chi T \qquad (1)$$

where ω_0 is the peak position of E^1_{2g} or A_{1g} mode at 0 K and χ is the first-order temperature coefficient. Temperature coefficients of Raman shifts were calculated and shown in Table S1. These values are close to other reports on few-layer WS_2 and MoS_2 nanosheets[S1-S3].

Table S1 | Temperature coefficients of Raman shift for WS_2 and MoS_2 nanosheets.

Nanosheets	E^1_{2g} ($cm^{-1}K^{-1}$)	A_{1g} ($cm^{-1}K^{-1}$)
WS_2	-0.005	-0.012
MoS_2	-0.016	-0.014

S2. Visible light responsive photocatalyst

Visible light responsive photocatalytic activity of as-synthesized WS_2 and MoS_2 nanosheets was demonstrated by treating Brilliant Green (BG) dye. 100 ppm 5 ml BG solutions containing nanosheets (1.4 mg each) were treated in dark to obtain the physical adsorption saturation. After that, the solutions were treated under visible light irradiation. Further reduction in BG concentration was directly associated with the photocatalytic activity of nanosheets. The concentration of dye was plotted as a function of time (treatment in light) using the following equation:

$$Ln(C) = Ln(C_0) - kt \qquad (2)$$

where C_0 and C are the dye concentrations at initial and any time t, and k is the reaction rate constant of the dye decomposition. Linear fits in Figure S2 indicate that the photocatalytic decomposition of BG dye is a first-order reaction for both WS_2 and MoS_2 nanosheets. The reaction rate constants were found to be 0.22 and 0.17 h^{-1} (i.e., 3.67×10^{-3} and 2.83×10^{-3} min^{-1}) for WS_2 and MoS_2 nanosheets, respectively. Higher rate constant for WS_2 nanosheets indicates faster kinetics compared to MoS_2 nanosheets.

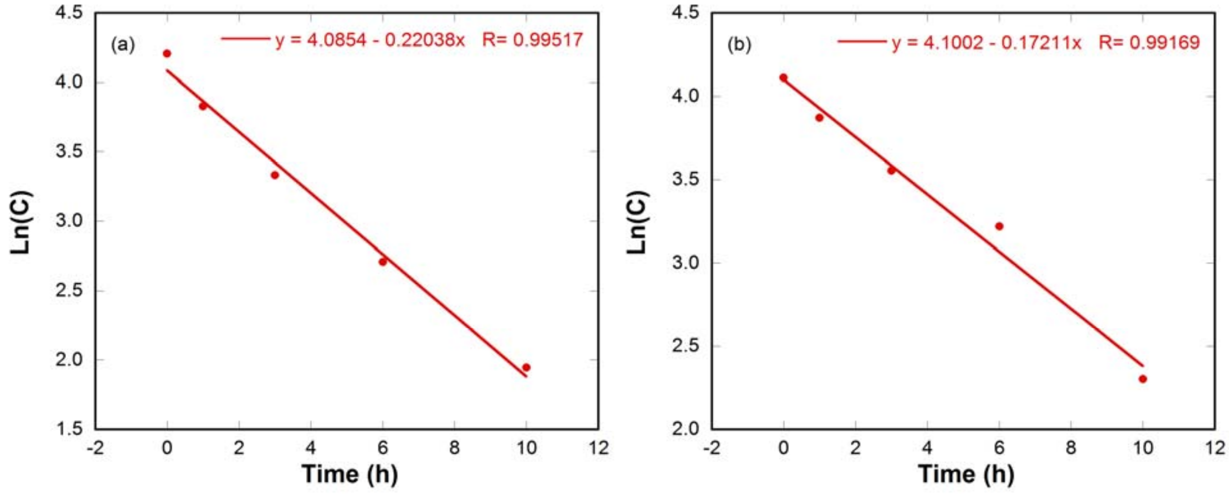

Figure S2 | Photocatalytic reaction kinetics. Concentration of BG dye as a function of time during photocatalytic decomposition under visible light irradiation followed by dark treatment with (a) WS_2 and (b) MoS_2 nanosheets in aqueous solution.

Reference

S1. Thripuranthaka, M., Kashid, R. V., Rout, C. S. & Late, D. J. Temperature dependent Raman spectroscopy of chemically derived few layer MoS_2 and WS_2 nanosheets. *Appl. Phys. Lett.* **104**, 081911 (2014).

S2. Peimyoo, N. *et al*. Thermal conductivity determination of suspended mono- and bilayer WS_2 by Raman spectroscopy. *Nano Res.*, DOI: 10.1007/s12274-014-0602-0 (2014).

S3. Yan, R. *et al*. Thermal conductivity of monolayer molybdenum disulfide obtained from temperature-dependent Raman spectroscopy. *ACS Nano* **8**, 986-993 (2014).
